# ELDER ABUSE IN SPECIAL POPULATIONS IN THE US: AMERICAN INDIANS AND ALASKA NATIVES AND THOSE LIVING IN THE RURAL SOUTH

**DOI:** 10.1093/geroni/igac059.1200

**Published:** 2022-12-20

**Authors:** Pamela B Teaster Teaster

**Affiliations:** Virginia Polytechnic Institute & State University, BLACKSBURG, Virginia, United States

## Abstract

The number of older adults who experience abuse, neglect, and exploitation as well as the complexities of the cases continue to increase, despite advances in prevention and intervention of the problem. At once a multifaceted problem that involves intertwining individual, relational, community, and societal contexts, issues related to elder abuse become even more complicated among special populations, which require a nuanced understanding and approach to prevention, detection, and intervention. This presentation is a compilation of the findings from studies of the mistreatment of older American Indians and Alaska Natives (AIANs) and adults living in the rural south, all populations that are understudied. Both research and practical policy and practice solutions that have the potential to resolve disparities in these populations will be discussed.

